# Trends and Disparities in Aspiration Pneumonitis Mortality With Co‐Listed Stroke Among US Adults, 1999–2024: A CDC WONDER Analysis

**DOI:** 10.1002/brb3.71739

**Published:** 2026-09-15

**Authors:** Mirza Ammar Arshad, Shiza Talib Butt, Wahhaj Munir, Muhammad Hassan Daniyal, Rohan Ahmad Daud, Warda Ikram, Rijas Ramish, Isbah Aman, Maryam Aslam, Hafiz Ahmad Masood, Asad Ali Ahmed Cheema

**Affiliations:** ^1^ Department of Medicine Rahbar Medical and Dental College Lahore Pakistan; ^2^ Department of Medicine Dow University of Health Sciences Karachi Pakistan; ^3^ Department of Medicine Fatima Jinnah Hospital Bahawalpur Pakistan; ^4^ Department of Medicine Rehman Medical Institute Peshawar Pakistan; ^5^ Department of Medicine Al‐Aleem Medical College Lahore Pakistan; ^6^ Department of Medicine Akhtar Saeed Medical and Dental College Lahore Pakistan; ^7^ Department of Medicine Ziauddin Medical College Karachi Pakistan; ^8^ Department of Medicine Quaid‐e‐Azam Medical College Bahawalpur Pakistan; ^9^ Department of Medicine International School of Medicine International University Kyrgyzstan Bishkek Kyrgyzstan

**Keywords:** aspiration pneumonitis, CDC WONDER, dysphagia, Joinpoint regression, mortality trends, stroke

## Abstract

**Background:**

Aspiration pneumonitis is a serious poststroke respiratory complication, yet contemporary US mortality trends involving aspiration pneumonitis as the underlying cause of death with co‐listed stroke remain unclear. We evaluated national trends and disparities in aspiration pneumonitis mortality with co‐listed stroke among US adults aged ≥ 25 years from 1999 to 2024.

**Methods:**

We conducted a retrospective, population‐based serial cross‐sectional analysis of CDC WONDER multiple cause of death data from 1999 through 2024. Deaths were included when aspiration pneumonitis (ICD‐10 J69.0) was the underlying cause and stroke (I60–I69) was a contributing cause among adults aged ≥ 25 years. Age‐adjusted mortality rates (AAMRs) per 100,000 population were standardized to the 2000 US population; age‐specific rates were crude. Joinpoint regression estimated annual percent changes (APCs) and average annual percent changes (AAPCs).

**Results:**

A total of 296,691 deaths were identified. The overall AAMR declined from 9.20 in 1999 to 3.55 in 2024 (AAPC, −3.66%; 95% CI, −4.34 to −2.97, *p* < 0.001). Mortality declined among men from 13.38 to 4.92 (AAPC, −3.85%) and women from 6.79 to 2.46 (AAPC, −3.92%; both *p* < 0.001). Mortality declined across all census regions, including Northeast (AAPC, −3.98%; *p* < 0.001), Midwest (AAPC, −3.76%; *p* < 0.001), South (AAPC, −3.93%; *p* < 0.001), and West (AAPC, −3.57%; *p* < 0.001), with the South having the higher AAMR (3.88) in 2024. Mortality remained higher in nonmetropolitan counties, with AAMR declining from 9.96 in 1999 to 4.42 in 2020 (AAPC, −4.05%; *p* < 0.001), compared with metropolitan counties, where the AAMR declined from 9.02 to 3.80 (AAPC, −4.31%; *p* < 0.001). Among non‐Hispanic Black adults, the 2014–2024 trend was stable (APC, 1.13%; *p* = 0.12). Rates increased after 2010 among adults aged 35–44 years (APC, 6.65%; *p* < 0.001), 45–54 years (APC, 4.70%; *p* < 0.001), and 55–64 years (APC, 3.96%; *p* < 0.001).

**Conclusion:**

Aspiration pneumonitis mortality with co‐listed stroke declined nationally, but progress was uneven, with persistent demographic, regional, and rural disparities.

## Graphical abstract

National mortality from aspiration pneumonitis with co‐listed stroke declined substantially among US adults from 1999–2024. However, persistent disparities by sex, age, race/ethnicity, region, and rurality, together with rising mortality in younger adults, highlight the need for equitable stroke prevention, dysphagia surveillance, and poststroke respiratory care.

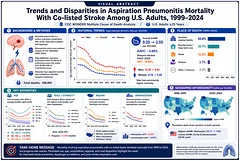

**Abbreviations**
AAMR: age‐adjusted mortality rate
AAPC: average annual percent change
APC: annual percent change
CDC WONDER: Centers for Disease Control and Prevention Wide‐ranging Online Data for Epidemiologic Research
CI: confidence interval
CMR
: crude mortality rate
ICD‐10: International Classification of Diseases, Tenth Revision
NCHS
: National Center for Health Statistics
NVSS
: National Vital Statistics System
SE
: standard error
STROBE
: Strengthening the Reporting of Observational Studies in Epidemiology

## 1 Introduction

Stroke remains a major cause of death and long‐term disability in the United States and is frequently complicated by swallowing dysfunction during both the acute and post‐acute phases of care (Lackland et al. 2014). Poststroke dysphagia is common after cerebrovascular injury and may impair airway protection, delay safe oral intake, and increase the risk of aspiration, particularly among patients with severe neurologic deficits, impaired consciousness, brainstem involvement, reduced mobility, or an ineffective cough reflex (Sherman et al. 2021; Martino et al. 2005). When gastric contents, oral secretions, or food material enter the lower respiratory tract, aspiration may result in chemical airway injury and aspiration pneumonitis. In this pathway, stroke is the usual underlying neurological condition, as the cerebrovascular injury can trigger dysphagia, loss of consciousness, coughing, and impaired airway protection following stroke, and then lead to aspiration pneumonitis as the subsequent respiratory complication (Sherman et al. 2021; Martino et al. 2005). Aspiration pneumonitis is not generally considered a cause of stroke. While respiratory symptoms of aspiration may be relatively mild, severe illness in the respiratory system, however, may theoretically lead to ischemic stroke as a result of systemic inflammation, endothelial dysfunction, hypoxemia, and activation of prothrombotic pathways (Cowan et al. 2016; Jung et al. 2024). However, such a reverse causality relationship cannot be confirmed with data from death certificates. The underlying cause of death was defined as aspiration pneumonitis and contributing cause of death was defined as stroke in the present study, as was prespecified on death certificates, but CDC WONDER database cannot determine the temporal or causal sequence for individual patients (Centers for Disease Control and Prevention 2024).

Poststroke dysphagia and aspiration‐related pulmonary complications are associated with substantial mortality, as shown by existing medical literature. A study involving 26,366 patients estimated that poststroke dysphagia was linked with approximately 4‐fold higher increase in risk of pneumonia and mortality (Banda et al. 2022). Epidemiological studies have similarly established the fact that poststroke dysphagia is a significant prognostic factor, resulting in higher short‐ and long‐term risks of pneumonia and death than in patients without dysphagia after stroke (Chang et al. 2022). However, most of the previous literature included poststroke pneumonia or general mortality exclusively using secondary databases, instead of deaths occurring with the specifically listed main cause of death as aspiration pneumonitis and the presence of stroke as a co‐listing condition.

Over the past several decades, stroke outcomes have improved through advances in acute stroke systems, organized stroke‐unit care, thrombolysis, mechanical thrombectomy, risk‐factor modification, and secondary prevention (Lackland et al. 2014; Ovbiagele et al. 2013). However, these gains have not been distributed equally across the US population. Prior studies have shown persistent disparities in stroke mortality and poststroke outcomes by age, sex, race and ethnicity, geographic region, and urbanization status (Ovbiagele et al. 2013; Howard and Howard 2020; Curtin 2024). Higher cerebrovascular mortality has been repeatedly observed in the Southern United States, particularly in states within the traditional Stroke Belt, while rural communities often face barriers to timely acute care, post‐acute rehabilitation, speech‐language pathology services, and longitudinal follow‐up (Howard and Howard 2020; Curtin 2024; Man et al. 2026). These same structural and clinical disparities may influence the recognition, prevention, and management of poststroke dysphagia and aspiration risk after discharge.

Existing national mortality studies have largely examined stroke mortality alone or broader categories of respiratory and infectious deaths, including pneumonia, respiratory failure, and sepsis‐related mortality (Ovbiagele et al. 2013; Freburger et al. 2025; Alam et al. 2026). However, national data specifically evaluating aspiration pneumonitis mortality in the setting of co‐listed cerebrovascular disease remain limited. This distinction is important because aspiration pneumonitis represents a clinically meaningful pathway through which neurologic impairment, swallowing dysfunction, frailty, and post‐acute care gaps may converge on fatal respiratory outcomes. By using a reproducible ICD‐10‐based definition and extending the analysis through 2024, the present study adds contemporary population‐level evidence regarding the demographic and geographic distribution of fatal aspiration pneumonitis among individuals with co‐listed stroke.

Therefore, we used the CDC WONDER multiple cause of death database to evaluate national trends in aspiration pneumonitis mortality with co‐listed stroke among US adults aged ≥ 25 years from 1999 through 2024. We examined temporal patterns overall and according to sex, age group, race and ethnicity, census region, state, and urbanization status. Temporal trends were assessed using Joinpoint regression to estimate APCs and AAPCs. The rationale for this study was to identify populations and geographic settings in which improvements in stroke management and aspiration prevention have not translated into comparable mortality reductions, thereby informing targeted vascular prevention, longitudinal dysphagia surveillance, rehabilitation, and post‐discharge care.

## 2 Methods

### 2.1 Study Design and Data Source

We conducted a retrospective, population‐based, cross‐sectional analysis of national mortality data obtained from the Centers for Disease Control and Prevention Wide‐ranging Online Data for Epidemiologic Research (CDC WONDER) multiple cause of death database. Mortality records were extracted for deaths occurring among US adults aged ≥ 25 years from January 1, 1999, to December 31, 2024. The multiple cause of death database contains death certificate‐based mortality data submitted to the National Vital Statistics System (NVSS) and includes one underlying cause of death and contributing causes of death coded according to the International Classification of Diseases, Tenth Revision (ICD‐10) (Centers for Disease Control and Prevention 2024; World Health Organization 2016).

The analysis included de‐identified, aggregate mortality data for all 50 US states and the District of Columbia. We used the multiple cause of death files to identify deaths involving aspiration pneumonitis and stroke according to prespecified ICD‐10 codes. Annual death counts, population denominators, crude mortality rates (CMRs), age‐adjusted mortality rates (AAMRs), standard errors (SE), and 95% confidence intervals (CI) were extracted where available. Data were stratified by calendar year, sex, age group, race and ethnicity, US Census region, state, urbanization status, and place of death according to variables available in CDC WONDER.

Because CDC WONDER provides publicly available, aggregate mortality data without individual‐level identifiers, institutional review board approval and informed consent were not required. The study was conducted and reported in accordance with the Strengthening the Reporting of Observational Studies in Epidemiology (STROBE) guidelines (von Elm et al. 2008).

### 2.2 Case Definition and ICD‐10 Codes

Deaths were identified using ICD‐10 codes recorded on death certificates. Aspiration pneumonitis was identified using ICD‐10 code J69.0. Importantly, the broader ICD‐10 category J69 is titled “Pneumonitis due to solids and liquids,” and aspiration pneumonia due to aspirated food or vomit may also be coded within J69.0. Therefore, some coding overlap between aspiration pneumonitis and aspiration pneumonia may occur in death‐certificate data. Aspiration pneumonitis was retained as the study's operational ICD‐10 label based on the prespecified case definition. Stroke was identified using ICD‐10 codes I60–I69, which include subarachnoid hemorrhage, intracerebral hemorrhage, other nontraumatic intracranial hemorrhage, cerebral infarction, stroke not specified as hemorrhage or infarction, and other cerebrovascular diseases (World Health Organization 2016).

For the primary analysis, records were included when aspiration pneumonitis was listed as the underlying cause of death and stroke was listed as a contributing cause of death. This definition was selected to identify deaths in which aspiration pneumonitis represented the primary certified cause of death in the setting of co‐listed cerebrovascular disease. The same ICD‐10 case definition was applied consistently across all study years and subgroup analyses.

### 2.3 Study Population

The study population included US adults aged ≥ 25 years whose death certificates met the prespecified ICD‐10 case definition between January 1, 1999, and December 31, 2024. The minimum age of 25 years was applied consistently to the overall and subgroup analyses. Deaths from all 50 US states and the District of Columbia were eligible for inclusion.

### 2.4 Demographic and Geographic Variables

Mortality data were stratified by prespecified demographic and geographic characteristics available in CDC WONDER, including year of death, sex, age group, race and ethnicity, US Census region, state, urbanization status, and place of death (Centers for Disease Control and Prevention 2024).

Sex was categorized as male or female, as recorded on death certificates. The term “sex” was used rather than “gender” because CDC WONDER death‐certificate data report sex categories and do not capture gender identity (Centers for Disease Control and Prevention 2024). Age was categorized into seven groups: 25–34, 35–44, 45–54, 55–64, 65–74, 75–84, and ≥ 85 years. Age‐specific analyses were reported using CMRs because each rate represented mortality within a defined age stratum (Anderson and Rosenberg 1998).

Race and ethnicity were categorized according to CDC WONDER/National Center for Health Statistics (NCHS) classifications as Hispanic or Latino, non‐Hispanic White, non‐Hispanic Black or African American, non‐Hispanic American Indian or Alaska Native, and non‐Hispanic Asian or Pacific Islander (Centers for Disease Control and Prevention 2024). Because racial and ethnic classifications differ across CDC WONDER database eras, Asian and Native Hawaiian or Other Pacific Islander categories from the 2018–2024 database were combined where necessary to maintain consistency with the 1999–2020 classification (Centers for Disease Control and Prevention 2024).

US Census regions were classified as Northeast, Midwest, South, and West. State‐level analyses included all 50 US states and the District of Columbia. Urbanization status was classified using the 2013 NCHS Urban–Rural Classification Scheme for Counties (Ingram and Franco 2013). For this analysis, counties were grouped as metropolitan and nonmetropolitan. Urbanization analyses were restricted to 1999–2020 period because urbanization categories were not available in the same format for the full 1999–2024 study period (Ingram and Franco 2013).

Place of death was categorized according to CDC WONDER death‐certificate classifications, including medical facility inpatient, medical facility outpatient or emergency department, dead on arrival, decedent's home, hospice facility, nursing home or long‐term care facility, and other or unknown place of death. Place‐of‐death results were reported as death counts and proportions rather than population‐based mortality rates because population denominators are not available for place‐of‐death categories.

### 2.5 Outcome Measures

The primary outcome was the annual AAMR for deaths in which aspiration pneumonitis was listed as the underlying cause of death and stroke was listed as a contributing cause of death. Mortality rates were expressed per 100,000 population. AAMRs were calculated using the direct standardization method and standardized to the 2000 US standard population (Anderson and Rosenberg 1998).

AAMRs were used for the overall analysis and for analyses stratified by sex, race and ethnicity, US Census region, state, and urbanization status (Anderson and Rosenberg 1998). CMRs were used for age‐specific analyses because each estimate represented mortality within a defined age stratum rather than across the full age distribution. Secondary outcomes included annual death counts, subgroup‐specific mortality rates, annual percent change, and average annual percent change (Centers for Disease Control and Prevention 2024).

For state‐level analyses, pooled age‐adjusted mortality rates were calculated across the study period to compare geographic variation in mortality burden. For place‐of‐death analyses, results were summarized using death counts and proportions rather than mortality rates because population denominators are not available for place‐of‐death categories in CDC WONDER (Centers for Disease Control and Prevention 2024).

### 2.6 Data Suppression and Reliability

In accordance with CDC WONDER confidentiality policies, death counts based on fewer than 10 deaths were suppressed and were not displayed in the database output (Centers for Disease Control and Prevention 2024). Suppressed values were not estimated, imputed, or treated as zero. Subgroup‐specific rates based on suppressed or sparse death counts were excluded from formal trend modeling where appropriate.

Rates designated as unreliable by CDC WONDER were interpreted cautiously. This was particularly relevant for smaller demographic subgroups and younger age categories, where annual death counts may be low and year‐to‐year variation may be unstable. When suppression or instability affected a subgroup, findings were reported descriptively rather than overinterpreted (Centers for Disease Control and Prevention 2024).

For Joinpoint regression, only annual rates with available estimates and corresponding standard errors or confidence intervals were used (National Cancer Institute 2024). Suppressed cells and unreliable estimates were excluded from trend modeling to avoid generating misleading annual percent change or average annual percent change estimates. These suppression rules were applied consistently across overall and subgroup‐specific analyses.

### 2.7 Statistical Analysis

Descriptive analyses were performed to summarize annual death counts, population denominators, CMRs, AAMRs, SE, and 95% CIs. Mortality rates were expressed per 100,000 population. AAMRs were calculated for overall trends and for analyses stratified by sex, race and ethnicity, US Census region, state, and urbanization status using the 2000 US standard population (Anderson and Rosenberg 1998). CMRs were used for age‐specific analyses because each estimate represented mortality within a defined age stratum.

Temporal trends were evaluated using the Joinpoint Regression Program, version 6.0.1, developed by the National Cancer Institute (National Cancer Institute 2024). Joinpoint regression was used to fit log‐linear models to annual mortality rates and identify calendar years in which the slope of the trend changed significantly. The number of joinpoints was allowed to range from 0 to 4. Model selection was based on permutation testing, with an overall significance level of 0.05 (National Cancer Institute 2024).

For each identified trend segment, annual percent change and corresponding 95% confidence intervals were calculated. Average annual percent change was calculated over the full study period from 1999 through 2024 to summarize the overall direction and magnitude of change (Centers for Disease Control and Prevention 2024). Trends were classified as increasing or decreasing when the 95% confidence interval for the annual percent change or average annual percent change did not include zero (National Cancer Institute 2024). Trends were considered stable when the corresponding 95% confidence interval crossed zero. Statistical significance was assessed at *p* < 0.05.

Numerical differences in mortality rates or average annual percent change values between subgroups were interpreted descriptively and were not described as statistically different unless supported by formal comparative testing. Urbanization‐specific trend analyses were restricted to 1999–2020 because urbanization data were not available in a comparable format for the full study period. Place‐of‐death analyses were summarized using death counts and proportions and were not modeled as population‐based mortality rates.

### 2.8 Ethical Considerations

This study used publicly available, de‐identified, aggregate mortality data obtained from the CDC WONDER multiple cause of death database (Centers for Disease Control and Prevention 2024). The database does not provide individual‐level identifiers, protected health information, or patient‐level clinical records. Therefore, institutional review board approval and informed consent were not required.

Because the analysis was based entirely on aggregate death‐certificate data, the findings were interpreted as population‐level mortality patterns rather than individual‐level clinical associations. The study was reported in accordance with the Strengthening the Reporting of Observational Studies in Epidemiology guidelines (von Elm et al. 2008).

## 3 Results

### 3.1 Overall Trends

**FIGURE 1:**
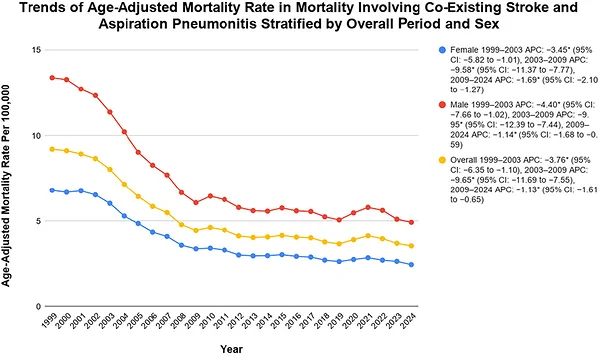
Overall and sex‐specific trends in aspiration pneumonitis mortality with co‐listed stroke, 1999–2024.

Between 1999 and 2024, 296,691 deaths occurred among US adults aged ≥ 25 years in which aspiration pneumonitis was listed as the underlying cause of death and stroke was listed as a contributing cause. Annual deaths decreased from 16,144 in 1999 to 10,050 in 2024. During the study period, the overall AAMR declined from 9.20 to 3.55 per 100,000 population. Joinpoint regression demonstrated a significant overall decline, with an AAPC of −3.66% (95% CI, −4.34 to −2.97). The decline occurred in three segments: 1999–2003 (APC, −3.76%; 95% CI, −6.35 to −1.10), 2003–2009 (APC, −9.65%; 95% CI, −11.69 to −7.55), and 2009–2024 (APC, −1.13%; 95% CI, −1.61 to −0.65). Thus, the steepest decline occurred from 2003 to 2009, followed by slower improvement through 2024 (Figure 1; Table 1; Tables S1 and S2).

**TABLE 1:** Annual and average annual percent changes in aspiration pneumonitis mortality with co‐listed stroke among US adults, 1999–2024.

Cohort	Segment	Lower endpoint	Upper endpoint	APC	Lower CI	Upper CI	AAPC (95% CI)
**Gender**							
Female	1	1999	2003	−3.4462*	−5.8222	−1.0102	−3.9203* (−4.5214 to −3.3154)
Female	2	2003	2009	−9.5842*	−11.3668	−7.7658	
Female	3	2009	2024	−1.6858*	−2.1035	−1.2663	
Male	1	1999	2003	−4.3983*	−7.6619	−1.0193	−3.8452* (−4.6702 to −3.013)
Male	2	2003	2009	−9.9512*	−12.3922	−7.4421	
Male	3	2009	2024	−1.1364*	−1.6797	−0.59	
Overall	1	1999	2003	−3.7575*	−6.3462	−1.0973	−3.6607* (−4.3419 to −2.9747)
Overall	2	2003	2009	−9.6460*	−11.6909	−7.5537	
Overall	3	2009	2024	−1.1306*	−1.6135	−0.6452	
**Census region**							
Northeast	1	1999	2003	−3.6218*	−7.0427	−0.0751	−3.9832* (−4.898 to −3.0595)
Northeast	2	2003	2009	−9.5798*	−12.3347	−6.7383	
Northeast	3	2009	2024	−1.7472*	−2.4149	−1.0749	
Midwest	1	1999	2003	−2.5623	−5.7287	0.7104	−3.7630* (−4.5694 to −2.9498)
Midwest	2	2003	2009	−8.6968*	−11.0981	−6.2306	
Midwest	3	2009	2024	−2.0401*	−2.6157	−1.461	
South	1	1999	2003	−4.7880*	−6.6546	−2.8841	−3.9316* (−4.7601 to −3.0958)
South	2	2003	2008	−11.3781*	−13.4722	−9.2334	
South	3	2008	2015	−2.5457*	−3.9302	−1.1412	
South	4	2015	2020	2.1035	−0.5978	4.8781	
South	5	2020	2024	−3.1031*	−5.4208	−0.7287	
West	1	1999	2003	−2.539	−6.0541	1.1077	−3.5720* (−4.5247 to −2.6098)
West	2	2003	2008	−10.1276*	−13.7337	−6.3707	
West	3	2008	2024	−1.6891*	−2.2118	−1.1637	
**Races**							
NH American Indian or Alaska Native	1	1999	2024	−4.5305*	−5.3941	−3.659	−4.5305* (−5.3941 to −3.659)
NH Asian or Pacific Islander	1	1999	2013	−6.5479*	−7.2397	−5.851	−4.2764* (−4.7985 to −3.7514)
NH Asian or Pacific Islander	2	2013	2024	−1.3053*	−2.2086	−0.3935	
NH Black or African American	1	1999	2003	−3.493	−8.0906	1.3346	−3.1454* (−4.7915 to −1.4709)
NH Black or African American	2	2003	2008	−11.1041*	−16.1051	−5.8051	
NH Black or African American	3	2008	2014	−2.9695	−7.3054	1.5693	
NH Black or African American	4	2014	2024	1.1325	−0.288	2.5733	
NH White	1	1999	2003	−3.6926*	−6.2618	−1.0531	−3.7615* (−4.4401 to −3.0781)
NH White	2	2003	2009	−9.4893*	−11.5415	−7.3895	
NH White	3	2009	2024	−1.3890*	−1.8676	−0.9079	
Hispanics or Latino	1	1999	2008	−8.6216*	−10.1372	−7.0805	−3.7703* (−4.417 to −3.1191)
Hispanics or Latino	2	2008	2024	−0.9290*	−1.5258	−0.3287	
**Urbanization**							
Metropolitan	1	1999	2003	−4.1292*	−6.2794	−1.9296	−4.3080* (−4.9614 to −3.6501)
Metropolitan	2	2003	2009	−9.6869*	−11.3396	−8.0033	
Metropolitan	3	2009	2020	−1.3072*	−1.8922	−0.7187	
Nonmetropolitan	1	1999	2003	−2.611	−5.3142	0.1693	−4.0495* (−4.9217 to −3.1693)
Nonmetropolitan	2	2003	2008	−8.9954*	−11.8007	−6.1008	
Nonmetropolitan	3	2008	2020	−2.3955*	−3.0663	−1.7201	
**10‐year age group**							
35–44 years	1	1999	2010	−1.7567	−4.3969	0.9563	2.8660* (1.3405 to 4.4145)
35–44 years	2	2010	2024	6.6502*	4.6923	8.6447	
45–54 years	1	1999	2010	−2.2827*	−4.3295	−0.192	1.5679* (0.468 to 2.6798)
45–54 years	2	2010	2024	4.6994*	3.4293	5.9851	
55–64 years	1	1999	2010	−5.2801*	−6.9406	−3.5901	−0.2094 (−1.1258 to 0.7155)
55–64 years	2	2010	2024	3.9644*	2.8678	5.0728	
65–74 years	1	1999	2003	−3.6999	−8.2098	1.0316	−2.6408* (−3.8321 to −1.4348)
65–74 years	2	2003	2009	−10.2990*	−13.8887	−6.5596	
65–74 years	3	2009	2024	0.8963*	0.1627	1.6353	
75–84 years	1	1999	2003	−4.0027*	−6.2628	−1.6881	−4.3248* (−4.9717 to −3.6734)
75–84 years	2	2003	2008	−10.4832*	−12.9688	−7.9266	
75–84 years	3	2008	2024	−2.3967*	−2.7891	−2.0028	
85+ years	1	1999	2003	−3.5127*	−5.8212	−1.1477	−4.3847* (−4.978 to −3.7877)
85+ years	2	2003	2009	−9.8294*	−11.6098	−8.0131	
85+ years	3	2009	2024	−2.3526*	−2.7696	−1.9337	

*Indicates that the Annual Percentage Change (APC) is significantly different from zero at α = 0.05.

### 3.2 Mortality Trends Stratified by Sex

From 1999 to 2024, men consistently had higher AAMRs than women. Among men, the AAMR declined from 13.38 to 4.92 per 100,000 population, while among women, it declined from 6.79 to 2.46 per 100,000 population. Joinpoint analysis showed significant declines in both groups. Among men, the AAMR declined from 1999 to 2003 (APC, −4.40%; 95% CI, −7.66 to −1.02), declined more steeply from 2003 to 2009 (APC, −9.95%; 95% CI, −12.39 to −7.44), and continued to decline from 2009 to 2024 (APC, −1.14%; 95% CI, −1.68 to −0.59), with an overall AAPC of −3.85% (95% CI, −4.67 to −3.01). Among women, corresponding APCs were −3.45% (95% CI, −5.82 to −1.01), −9.58% (95% CI, −11.37 to −7.77), and −1.69% (95% CI, −2.10 to −1.27), with an overall AAPC of −3.92% (95% CI, −4.52 to −3.32). Despite long‐term improvement, men continued to have approximately twice the mortality rate of women by 2024 (Figure 1; Table 1; Tables S1 and S2).

### 3.3 Mortality Trends Stratified by Race and Ethnicity

Mortality trends varied by race and ethnicity. Non‐Hispanic Black adults had the highest AAMR at the beginning of the study period, at 12.51 per 100,000 population in 1999. Among non‐Hispanic Black adults, the AAMR declined nonsignificantly from 1999 to 2003 (APC, −3.49%; 95% CI, −8.09 to 1.33), declined significantly from 2003 to 2008 (APC, −11.10%; 95% CI, −16.11 to −5.81), declined nonsignificantly from 2008 to 2014 (APC, −2.97%; 95% CI, −7.31 to 1.57), and was stable from 2014 to 2024 (APC, 1.13%; 95% CI, −0.29 to 2.57), with an overall AAPC of −3.15% (95% CI, −4.79 to −1.47).

**FIGURE 2:**
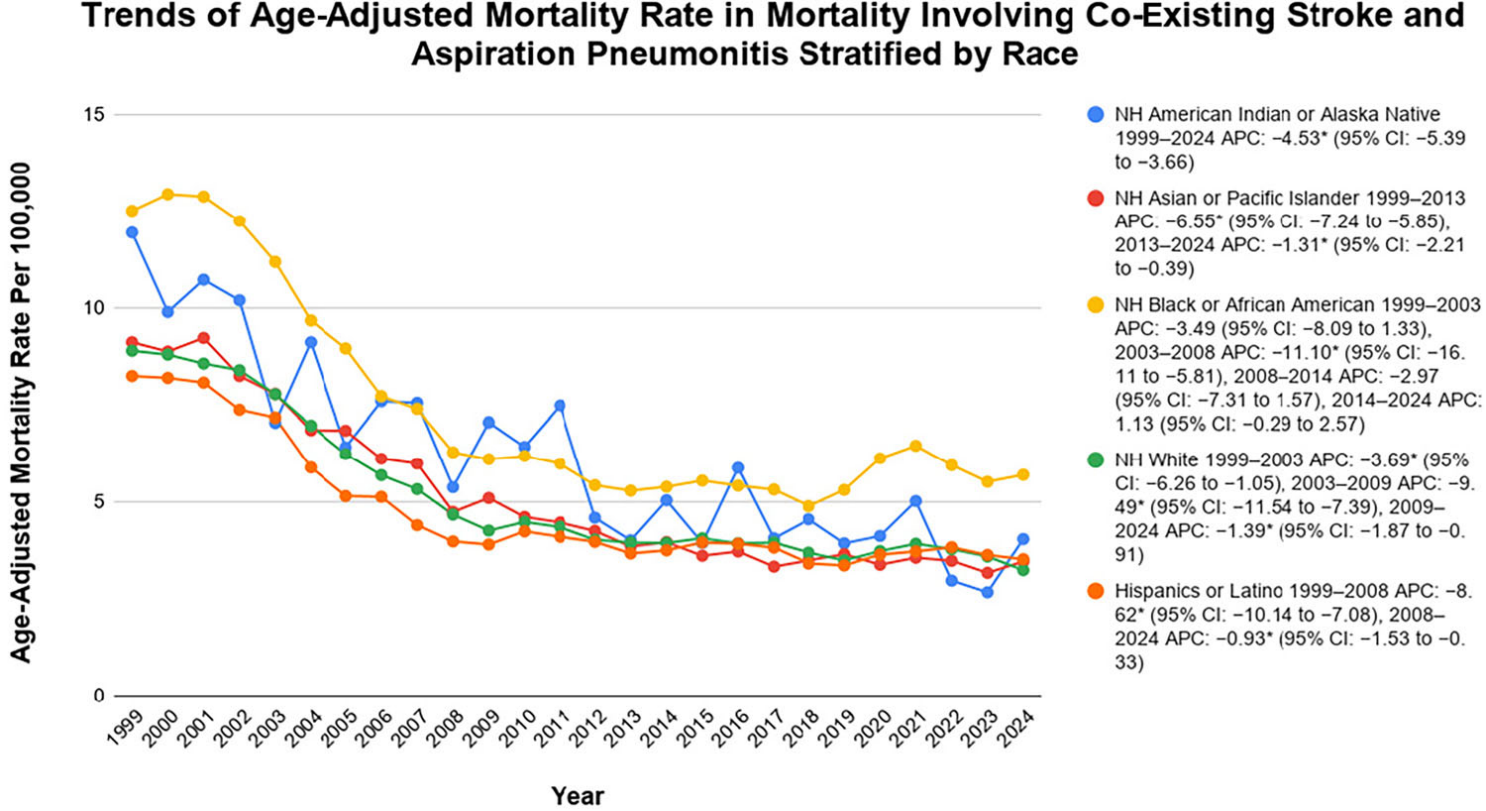
Race‐ and ethnicity‐specific trends in aspiration pneumonitis mortality with co‐listed stroke, 1999–2024.

Among non‐Hispanic White adults, the AAMR declined from 1999 to 2003 (APC, −3.69%; 95% CI, −6.26 to −1.05), 2003 to 2009 (APC, −9.49%; 95% CI, −11.54 to −7.39), and 2009 to 2024 (APC, −1.39%; 95% CI, −1.87 to −0.91), with an AAPC of −3.76% (95% CI, −4.44 to −3.08). AAMR among Hispanic or Latino adults declined from 1999 to 2008 (APC, −8.62%; 95% CI, −10.14 to −7.08) and 2008 to 2024 (APC, −0.93%; 95% CI, −1.53 to −0.33), with an AAPC of −3.77% (95% CI, −4.42 to −3.12). The rate for non‐Hispanic Asian or Pacific Islander adults declined from 1999 to 2013 (APC, −6.55%; 95% CI, −7.24 to −5.85) and 2013 to 2024 (APC, −1.31%; 95% CI, −2.21 to −0.39), with an AAPC of −4.28% (95% CI, −4.80 to −3.75). Non‐Hispanic American Indian or Alaska Native adults showed a single declining trend, with an APC and AAPC of −4.53% (95% CI, −5.39 to −3.66) (Figure 2; Table 1; Tables S1 and S3).

### 3.4 Mortality Trends Stratified by Census Region

Mortality declined across all US Census regions. The South had the highest AAMR throughout the study period, declining from 10.62 per 100,000 population in 1999 to 3.88 in 2024. In the South, rates declined from 1999 to 2003 (APC, −4.79%; 95% CI, −6.65 to −2.88), 2003 to 2008 (APC, −11.38%; 95% CI, −13.47 to −9.23), and 2008 to 2015 (APC, −2.55%; 95% CI, −3.93 to −1.14). This was followed by a stable interval from 2015 to 2020 (APC, 2.10%; 95% CI, −0.60 to 4.88) and a renewed decline from 2020 to 2024 (APC, −3.10%; 95% CI, −5.42 to −0.73), with an AAPC of −3.93% (95% CI, −4.76 to −3.10).

**FIGURE 3:**
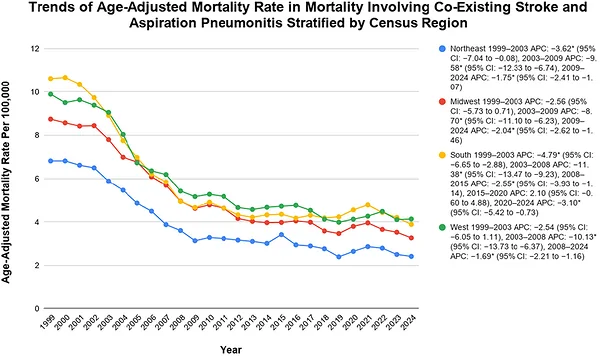
Census region‐specific trends in aspiration pneumonitis mortality with co‐listed stroke, 1999–2024.

The Northeast showed an AAPC of −3.98% (95% CI, −4.90 to −3.06), with APCs of −3.62% from 1999 to 2003, −9.58% from 2003 to 2009, and −1.75% from 2009 to 2024. The Midwest had an AAPC of −3.76% (95% CI, −4.57 to −2.95), with APCs of −2.56% from 1999 to 2003, −8.70% from 2003 to 2009, and −2.04% from 2009 to 2024. The West had an AAPC of −3.57% (95% CI, −4.52 to −2.61), with APCs of −2.54% from 1999 to 2003, −10.13% from 2003 to 2008, and −1.69% from 2008 to 2024 (Figure 3; Table 1; Tables S1 and S4).

### 3.5 Mortality Trends Stratified by Urbanization

Urbanization‐specific analyses were restricted to 1999–2020 because comparable data were not available through 2024. Mortality declined in both metropolitan and nonmetropolitan counties, although nonmetropolitan counties had consistently higher AAMRs. In 1999, the AAMR was 9.96 per 100,000 population in nonmetropolitan counties and 9.02 per 100,000 population in metropolitan counties. By 2020, these rates declined to 4.42 and 3.80 per 100,000 population, respectively.

**FIGURE 4:**
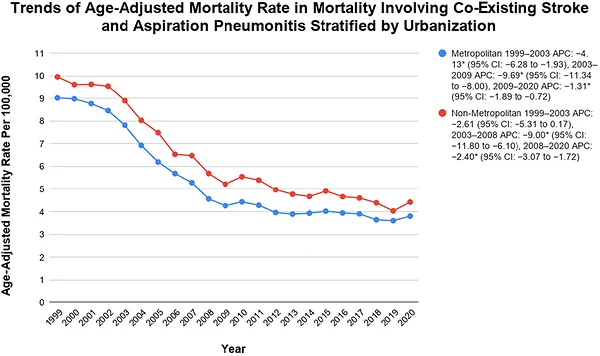
Urbanization‐specific trends in aspiration pneumonitis mortality with co‐listed stroke, 1999–2020.

Among metropolitan counties, AAMRs declined from 1999 to 2003 (APC, −4.13%; 95% CI, −6.28 to −1.93), from 2003 to 2009 (APC, −9.69%; 95% CI, −11.34 to −8.00), and from 2009 to 2020 (APC, −1.31%; 95% CI, −1.89 to −0.72), with an AAPC of −4.31% (95% CI, −4.96 to −3.65). Among nonmetropolitan counties, the AAMR declined nonsignificantly from 1999 to 2003 (APC, −2.61%; 95% CI, −5.31 to 0.17), followed by significant declines from 2003 to 2008 (APC, −9.00%; 95% CI, −11.80 to −6.10) and 2008 to 2020 (APC, −2.40%; 95% CI, −3.07 to −1.72), with an AAPC of −4.05% (95% CI, −4.92 to −3.17) (Figure 4; Table 1; and Tables S1 and S5).

### 3.6 Mortality Trends Stratified by Age

Age‐specific analyses were reported using CMR. Mortality trends differed markedly across age groups, with recent increases among younger and middle‐aged adults but continued long‐term declines among older adults. Among adults aged 35–44 years, the rate declined nonsignificantly from 1999 to 2010 (APC, −1.76%; 95% CI, −4.40 to 0.96), followed by a significant increase from 2010 to 2024 (APC, 6.65%; 95% CI, 4.69 to 8.64), with an AAPC of 2.87% (95% CI, 1.34 to 4.41). Adults aged 45–54 years showed a decline from 1999 to 2010 (APC, −2.28%; 95% CI, −4.33 to −0.19), followed by an increase from 2010 to 2024 (APC, 4.70%; 95% CI, 3.43 to 5.99), with an AAPC of 1.57% (95% CI, 0.47 to 2.68). Adults aged 55–64 years showed a rate that declined from 1999 to 2010 (APC, −5.28%; 95% CI, −6.94 to −3.59), then increased from 2010 to 2024 (APC, 3.96%; 95% CI, 2.87 to 5.07), with a non‐significant AAPC of −0.21% (95% CI, −1.13 to 0.72).

**FIGURE 5:**
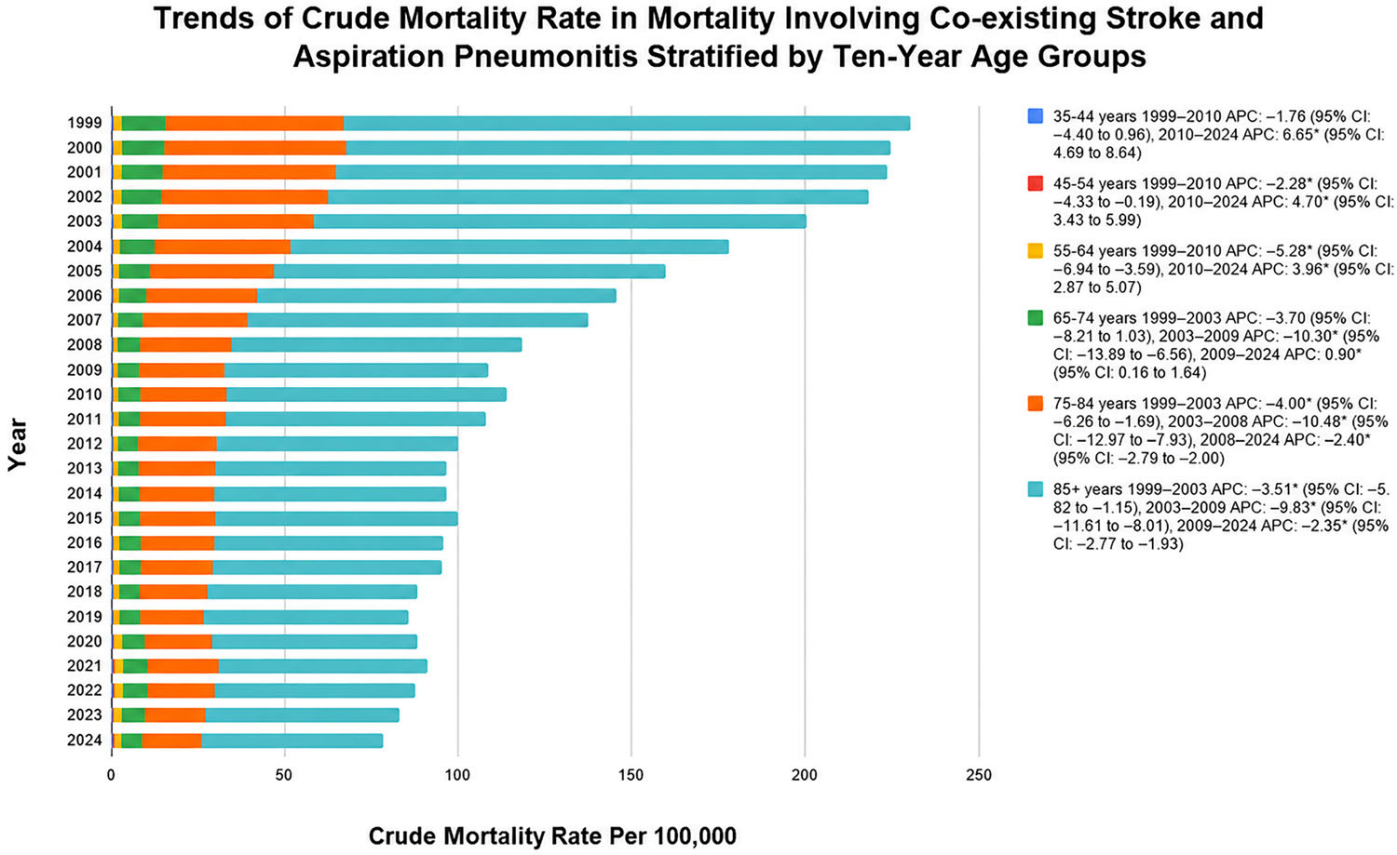
Age‐specific trends in aspiration pneumonitis mortality with co‐listed stroke, 1999–2024.

Among adults aged 65–74 years, rates declined from 1999 to 2003 (APC, −3.70%; 95% CI, −8.21 to 1.03) and from 2003 to 2009 (APC, −10.30%; 95% CI, −13.89 to −6.56), followed by an increase from 2009 to 2024 (APC, 0.90%; 95% CI, 0.16 to 1.64), with an AAPC of −2.64% (95% CI, −3.83 to −1.43). In contrast, adults aged 75–84 years showed sustained declines across 1999–2003 (APC, −4.00%; 95% CI, −6.26 to −1.69), 2003–2008 (APC, −10.48%; 95% CI, −12.97 to −7.93), and 2008–2024 (APC, −2.40%; 95% CI, −2.79 to −2.00), with an AAPC of −4.32% (95% CI, −4.97 to −3.67). Adults aged ≥ 85 years also declined across 1999–2003 (APC, −3.51%; 95% CI, −5.82 to −1.15), 2003–2009 (APC, −9.83%; 95% CI, −11.61 to −8.01), and 2009–2024 (APC, −2.35%; 95% CI, −2.77 to −1.93), with an AAPC of −4.38% (95% CI, −4.98 to −3.79) (Figure 5; Table 1; Tables S1 and S6).

### 3.7 Mortality Trends Stratified by Place of Death and State

**FIGURE 6:**
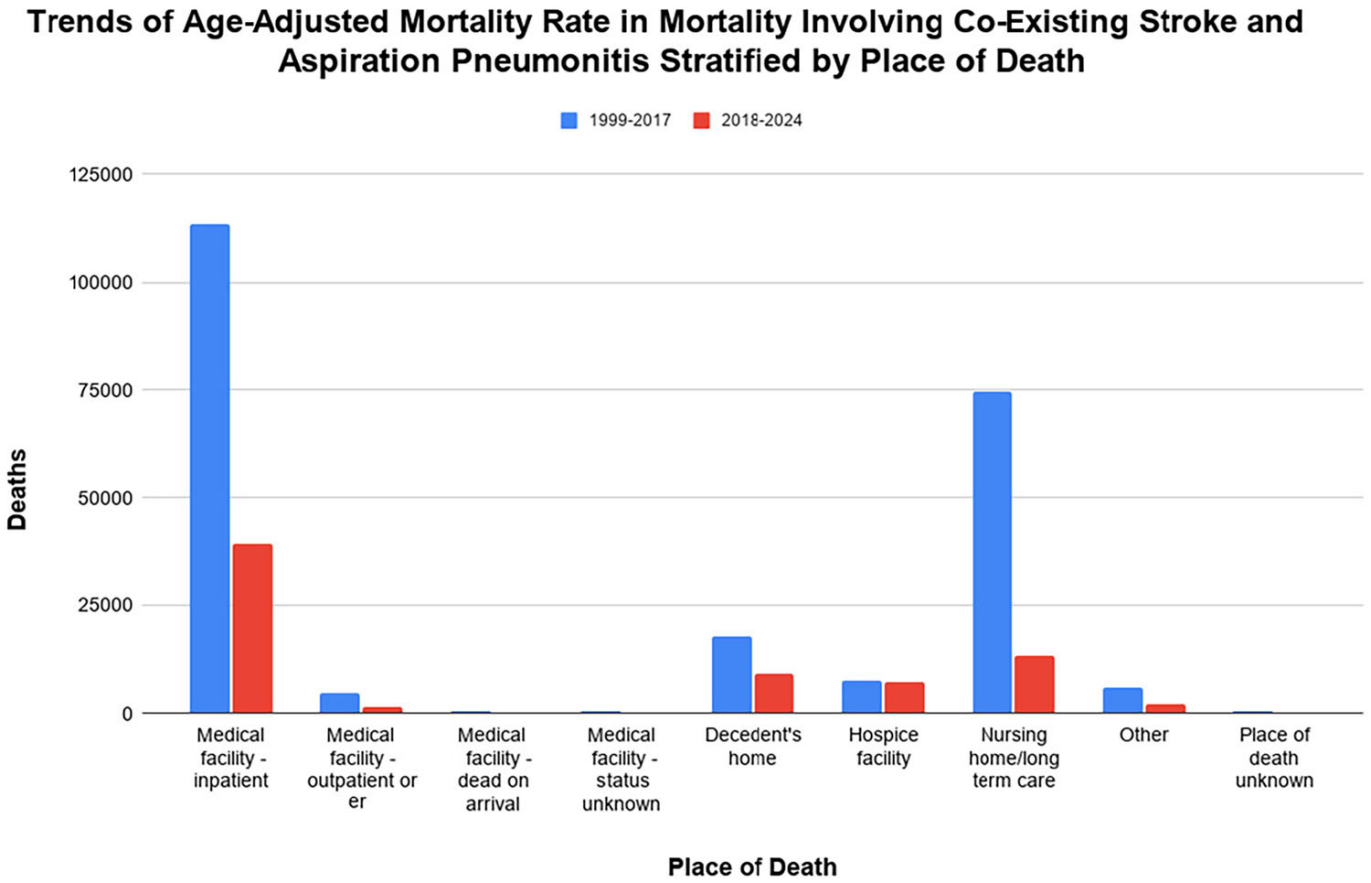
Place‐of‐death distribution for aspiration pneumonitis mortality with co‐listed stroke.

Most deaths occurred in institutional care settings. Hospital inpatient deaths accounted for 152,417 deaths, representing 51.3% of all deaths, followed by nursing home or long‐term care facility deaths, with 87,803 deaths or 29.6%. Deaths at home accounted for 26,719 deaths or 9.0%, and hospice facility deaths accounted for 14,750 deaths or 5.0%. Smaller proportions occurred in medical facility outpatient or emergency department settings (5,943 deaths; 2.0%), other locations (8,014 deaths; 2.7%), dead‐on‐arrival settings (390 deaths; 0.1%), medical facility status unknown (333 deaths; 0.1%), and unknown place of death (557 deaths; 0.2%) (Figure 6; Tables S1 and S7).

**FIGURE 7:**
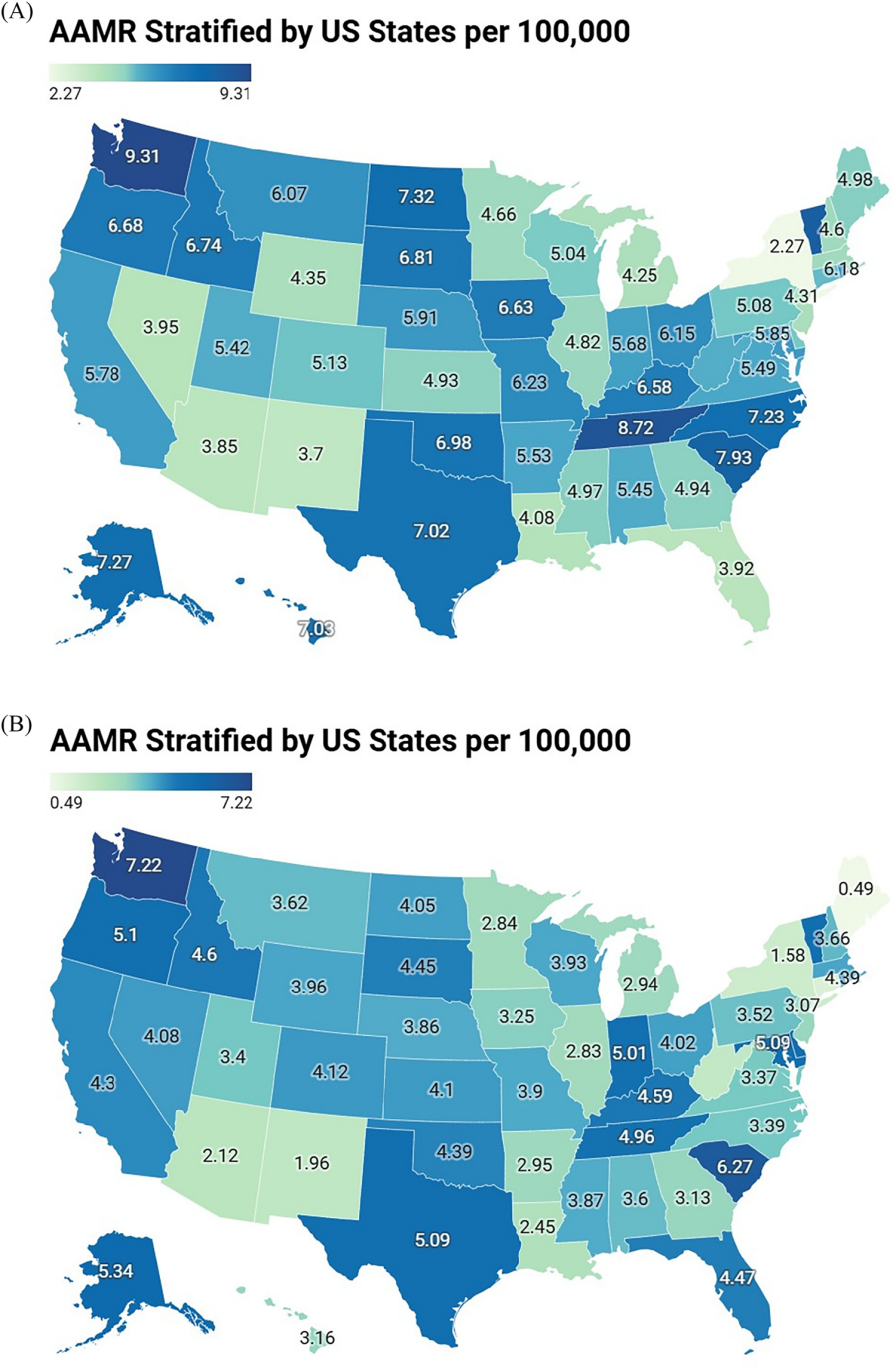
(A) State‐level age‐adjusted mortality rates for aspiration pneumonitis mortality with co‐listed stroke, 1999–2020. (B) State‐level age‐adjusted mortality rates for aspiration pneumonitis mortality with co‐listed stroke, 2021–2024.

State‐level analyses showed substantial geographic variation. During 1999–2020, Washington had the highest AAMR at 9.31 per 100,000 population, followed by Tennessee at 8.72, Vermont at 8.17, South Carolina at 7.93, and the District of Columbia at 7.78. The lowest AAMRs were observed in New York at 2.27, New Mexico at 3.70, Arizona at 3.85, Florida at 3.92, and Nevada at 3.95. During 2021–2024, Washington again had the highest AAMR at 7.22, followed by the District of Columbia at 6.63, South Carolina at 6.27, Alaska at 5.34, Oregon at 5.10, Texas at 5.09, and Maryland at 5.09. The lowest AAMRs were observed in Maine at 0.49, New York at 1.58, New Mexico at 1.96, Arizona at 2.12, and Louisiana at 2.45 per 100,000 population (Figure 7A,B; Table S8).

## 4 Discussion

In this national CDC WONDER analysis of US adults aged ≥ 25 years, aspiration pneumonitis mortality with co‐listed stroke declined substantially from 1999 to 2024. The overall AAMR decreased from 9.20 to 3.55 per 100,000 population, with the steepest decline observed between 2003 and 2009. However, this favorable national trend concealed important subgroup differences. The pace of decline slowed after 2009; men continued to have nearly twice the mortality burden of women; younger and middle‐aged adults showed late‐period increases; non‐Hispanic Black adults experienced attenuation of earlier improvement; and mortality remained higher in the South and in nonmetropolitan counties. These findings suggest that although aspiration pneumonitis mortality in the setting of co‐listed stroke has improved nationally, progress has been uneven across demographic and geographic groups.

The early decline in mortality is consistent with broader improvements in stroke prevention, acute stroke systems, and poststroke care. Stroke mortality in the United States declined substantially during the late 20th and early 21st centuries, likely reflecting better hypertension control, reductions in smoking, lipid management, organized stroke care, and advances in acute treatment (Lackland et al. 2014; Ovbiagele et al. 2013). In the context of aspiration pneumonitis, this decline is clinically plausible because improvements in stroke care may reduce the severity of neurologic deficits that predispose patients to dysphagia, impaired cough, immobility, and aspiration. The increasing use of structured dysphagia screening may also have contributed. Dysphagia after stroke is strongly associated with pulmonary complications, and screening programs have been associated with improved clinical outcomes, including reduced pneumonia and mortality (Sherman et al. 2021; Martino et al. 2005). Therefore, the marked decline during the early study period may reflect combined gains in vascular prevention, acute stroke management, and aspiration‐risk recognition.

The overall drop may be partly attributed to changes in treatment modalities (Lackland et al. 2014; Ovbiagele et al. 2013). The use of stroke unit care, intravenous thrombolysis, and mechanical thrombectomy increased during the study period, with a corresponding improvement in survival and functional outcomes after acute stroke, which may have contributed to a decrease in the severity of neurological deficits related to dysphagia, ineffective cough, immobility, and impaired airway protection (Lackland et al. 2014; Ovbiagele et al. 2013). Other factors such as increased use of early standardized dysphagia screening and speech‐language pathology assessment, texture‐modified diets, feeding support, oral hygiene practices, and individualized nutritional support may also have contributed to decreased aspiration‐related pulmonary complications and mortality (Sherman et al. 2021; Martino et al. 2005). Dysphagia screening following stroke has been found to be associated with reduced risk of pneumonia and death, with the extent of the benefit dependent on the type of screening and subsequent management (Sherman et al. 2021). The observed trends might also have been affected by changes in the recognition of the diseases and in the coding of the cause of death (Centers for Disease Control and Prevention 2024; Son et al. 2017; Almirall et al. 2021). Aspiration pneumonitis is a noninfectious chemical lung injury due to the aspiration of gastric contents, and aspiration pneumonia is an infectious process that is associated with the aspiration of bacteria‐containing material (Son et al. 2017; Almirall et al. 2021). However, both conditions often occur together in clinical practice and can be hard to distinguish, especially if the aspiration is not witnessed (Son et al. 2017; Almirall et al. 2021). Definitions of diagnosis have varied from study to study and across clinical situations, and deaths could be given alternative causes such as aspiration pneumonia, bacterial pneumonia, respiratory failure, or sepsis (Centers for Disease Control and Prevention 2024; Son et al. 2017; Almirall et al. 2021). This could, therefore, have changed over time with the increased awareness of poststroke aspiration, the increased availability of swallowing assessments, and the changing coding or certification processes in ICD‐10 (Sherman et al. 2021; Centers for Disease Control and Prevention 2024; Son et al. 2017; Almirall et al. 2021). Thus, the observed decrease is likely due to improved care and improved diagnostic and death certification practices.

The slowing of improvement after 2009 is clinically important. Although mortality continued to decline, the later trend segment showed only modest annual improvement, suggesting that earlier gains may have reached a plateau. This pattern is consistent with broader concerns that progress in US stroke and cardiovascular mortality has slowed in recent years, particularly in the setting of persistent cardiometabolic risk and widening geographic disparities (Anderson and Rosenberg 1998; Ingram and Franco 2013; National Cancer Institute 2024). Aspiration pneumonitis after stroke is unlikely to represent an isolated terminal event. Rather, it may reflect a continuum beginning with vascular risk exposure, followed by stroke severity, dysphagia, impaired airway protection, functional dependence, and the quality of post‐acute care (Martino et al. 2005; Martino et al. 2012). Because CDC WONDER does not include patient‐level clinical data, these pathways cannot be directly tested; however, the observed trend pattern supports the need to strengthen both stroke prevention and aspiration‐risk management beyond the acute hospital setting (Centers for Disease Control and Prevention 2024).

The age‐specific findings are particularly concerning. Mortality continued to decline among older adults, whereas adults aged 35–44, 45–54, and 55–64 years experienced increasing mortality after 2010 (Anderson and Rosenberg 1998). This divergence suggests that the overall national decline was driven largely by improvements among older adults, while younger and middle‐aged adults may represent an emerging high‐risk group. Rising cardiometabolic risk, including hypertension, diabetes, obesity, and dyslipidemia, may contribute to more severe or recurrent cerebrovascular disease at younger ages (Howard and Howard 2020; Vaughan et al. 2022). National data also show that stroke death rates among adults aged 45–64 years increased after earlier declines, with persistent differences by sex, region, and race and ethnicity (Curtin 2024). In the setting of aspiration pneumonitis, younger stroke survivors with residual dysphagia may face different vulnerabilities than older adults, including earlier discharge home, limited access to rehabilitation, inconsistent outpatient swallowing reassessment, and variable caregiver support. These findings support greater attention to dysphagia surveillance and post‐discharge follow‐up among younger and middle‐aged stroke survivors.

Men had consistently higher mortality than women throughout the study period. This excess burden may reflect higher vascular risk exposure, smoking history, ischemic heart disease, differences in stroke severity, and differences in care‐seeking behavior before and after stroke (Lackland et al. 2014; Ovbiagele et al. 2013). In aspiration‐related mortality, male risk may also be influenced by comorbidity clustering and reduced pulmonary reserve. Importantly, mortality declined in both men and women, suggesting that system‐level improvements may have benefited both groups. However, the persistent absolute gap indicates that prevention strategies should continue to emphasize vascular risk modification, early recognition of dysphagia, and structured poststroke respiratory monitoring among men.

Racial and ethnic patterns also deserve careful interpretation. Non‐Hispanic Black adults had the highest mortality burden early in the study period and showed attenuation of improvement in later years. This is consistent with broader evidence showing higher stroke burden, earlier onset of vascular risk factors, lower rates of blood pressure control, and persistent inequities in access to preventive and rehabilitative care among Black adults (Howard and Howard 2020; Vaughan et al. 2022; Carnethon et al. 2017). In this study, the late‐period trend among non‐Hispanic Black adults should not be interpreted as definitive evidence of worsening because some estimates may be affected by uncertainty; however, the pattern suggests that earlier gains were not sustained equally. These findings highlight the importance of addressing both upstream stroke prevention and downstream recovery resources, including access to speech‐language pathology, rehabilitation, and outpatient follow‐up.

Geographic findings further emphasize the role of place in poststroke respiratory mortality. The South had the highest mortality burden throughout the study period and experienced a period of relative stagnation despite the national decline. This aligns with the longstanding excess cerebrovascular mortality observed in the Stroke Belt and surrounding Southern states (Howard and Howard 2020). Higher burdens of hypertension‐related cardiovascular mortality have also been concentrated in Southern counties, particularly among middle‐aged adults (Vaughan et al. 2022). These regional patterns may reflect the combined influence of vascular risk burden, rurality, socioeconomic disadvantage, and variable access to stroke and rehabilitation services. The state‐level variation observed in this study should not be interpreted as evidence of causal state‐level effects, but it identifies jurisdictions where surveillance, prevention, coding review, and post‐acute care access may warrant closer evaluation.

Urbanization‐specific results add an important health‐system perspective. Nonmetropolitan counties had higher mortality than metropolitan counties from 1999 to 2020. Rural residence can affect multiple stages of stroke care, including time to acute treatment, access to certified stroke centers, availability of neurologists, rehabilitation intensity, speech‐language pathology services, transportation, and caregiver support (Vaughan et al. 2022; Aggarwal et al. 2021). Aspiration pneumonitis is particularly sensitive to these gaps because prevention requires repeated swallowing reassessments, diet modification, oral hygiene, feeding assistance, medication review, and early response to respiratory symptoms (Dziewas et al. 2021). A single inpatient swallow screen may be insufficient if dysphagia persists or worsens after discharge (Martino et al. 2005). Therefore, the rural excess supports the need for stronger poststroke transitions, tele‐rehabilitation, remote dysphagia monitoring, and standardized follow‐up pathways in nonmetropolitan communities.

The place‐of‐death distribution provides additional clinical insight. More than half of deaths occurred in inpatient medical facilities, and a substantial proportion occurred in nursing homes or long‐term care facilities. This distribution suggests that many decedents were medically complex, functionally dependent, or already receiving institutional care. It also identifies hospitals and long‐term care facilities as key settings for prevention. Standardized swallowing protocols, aspiration‐risk reassessment, oral hygiene programs, feeding assistance, medication review, and early recognition of respiratory deterioration may be particularly important in these environments (Martino et al. 2005; Dziewas et al. 2021). The long‐term care burden is especially relevant because dysphagia may persist long after the index stroke and may fluctuate with delirium, sedating medications, recurrent neurologic events, infection, or progressive frailty (Martino et al. 2005; González‐Fernández et al. 2013).

These findings have several clinical and public‐health implications. First, vascular risk prevention in younger and middle‐aged adults should remain a priority because aspiration pneumonitis mortality with co‐listed stroke is not confined to advanced age. Second, dysphagia screening should be viewed as part of a longitudinal care pathway rather than a single inpatient checklist. Patients discharged home after stroke may require structured reassessments, caregiver education, dietary guidance, and timely speech‐language pathology follow‐up. Third, high‐burden regions and nonmetropolitan communities may benefit from expanded telestroke networks, tele‐rehabilitation, and standardized discharge pathways. Fourth, racial equity efforts should address both upstream stroke prevention and downstream recovery resources because aspiration‐related mortality may reflect vulnerabilities across the entire care continuum.

This study has several strengths. It used a large, nationally representative mortality database covering all 50 US states and the District of Columbia over 26 years. The use of a reproducible ICD‐10 case definition allowed systematic identification of deaths in which aspiration pneumonitis was recorded as the underlying cause and stroke was listed as a contributing condition. Mortality rates were standardized to the 2000 US standard population where appropriate, and Joinpoint regression provided objective assessment of temporal changes. The analysis also evaluated multiple clinically relevant subgroups, including sex, age, race and ethnicity, US Census region, urbanization, state‐level variation, and place of death.

Several limitations should be considered. First, CDC WONDER relies on death‐certificate data, which may be affected by inaccurate cause‐of‐death documentation, changes in coding practices, and variation across clinicians, institutions, and states. Aspiration pneumonitis may be under‐recorded when deaths are attributed to pneumonia, sepsis, or respiratory failure without documentation of aspiration. In addition, because ICD‐10 code J69.0 may also be used for aspiration pneumonia in death‐certificate coding, some diagnostic overlap between aspiration pneumonitis and aspiration pneumonia cannot be excluded. This potential coding overlap should be considered when interpreting the findings (World Health Organization 2016; Son et al. 2017; Almirall et al. 2021). Second, the presence of stroke as a contributing cause does not establish that stroke directly caused aspiration pneumonitis. Third, the database does not provide information on stroke subtype, lesion location, neurologic severity, dysphagia severity, swallow‐screen timing, diet modification, feeding tube use, rehabilitation intensity, medications, comorbidities, socioeconomic status, insurance coverage, or health‐care access. Fourth, urbanization data were available only through 2020, limiting rural‐urban analyses for the most recent years. Fifth, smaller subgroups, particularly younger adults and some racial and ethnic groups, may be affected by suppressed counts or unstable annual rates. Finally, because this was an ecological mortality analysis, the observed patterns should be interpreted as population‐level associations rather than individual‐level causal relationships.

## 5 Conclusion

Aspiration pneumonitis mortality with co‐listed stroke declined substantially among US adults aged ≥ 25 years from 1999 to 2024, but persistent age, sex, racial, regional, and rural disparities remain. Targeted vascular prevention, longitudinal dysphagia surveillance, equitable rehabilitation, and improved post‐discharge care are needed to reduce this residual burden.

## Author Contributions

**Mirza Ammar Arshad**: conceptualization, data curation, formal analysis, project administration, software, validation, supervision, and writing – review and editing. **Shiza Talib Butt**: conceptualization, investigation, validation, writing – original draft, writing – review and editing. **Wahhaj Munir**: data acquisition, investigation, writing – original draft, writing – review and editing, and validation. **Muhammad Hassan Daniyal**: data acquisition, data curation, investigation, and writing – original draft. **Rohan Ahmad Daud**: data curation, investigation, and writing – original draft. **Warda Ikram**: formal analysis, validation, visualization, writing – original draft. **Rijas Ramish**: methodology, investigation, writing – original draft. **Isbah Aman**: investigation, literature review, writing – original draft. **Maryam Aslam**: visualization, writing – original draft. **Hafiz Ahmad Masood**: data curation and writing – original draft. **Asad Ali Ahmed Cheema**: supervision, project administration, validation, writing – review and editing.

## Funding

The authors have nothing to report.

## Ethics Statement

The authors have nothing to report.

## Consent

The authors have nothing to report.

## Conflicts of Interest

The authors declare no conflicts of interest.

## Supporting information




**Supplementary Information**: brb371739‐sup‐0001‐SuppMat.docx

## Data Availability

The data used in this study are publicly available from the Centers for Disease Control and Prevention Wide‐ranging Online Data for Epidemiologic Research multiple cause of death database. The datasets can be accessed through CDC WONDER. All data used in this analysis were aggregate and de‐identified.
