# Supplementary material for: Trends and Disparities in Aspiration Pneumonitis Mortality With Co‐Listed Stroke Among US Adults, 1999–2024: A CDC WONDER Analysis
Source: Brain Behav. 2026 Sep 15;16(9):e71739. doi: 10.1002/brb3.71739 (PMC13575627; doi:10.1002/brb3.71739)
Supplement: Supplementary file 1 [file BRB3-16-e71739-s001.docx]

**Supplementary Material Legend**

**Table S1.** Total deaths and percentage distribution by study variables

**Table S2.** Annual AAMR by overall mortality and sex, 1999-2024

**Table S3.** Annual AAMR by race and ethnicity, 1999–2024

**Table S4.** Annual AAMR by U.S. Census region, 1999–2024

**Table S5.** Annual AAMR by urbanization status, 1999–2020

**Table S6.** Annual crude mortality rates by age group, 1999–2024

**Table S7.** Place-of-death distribution, 1999–2024

**Table S8.** State-level AAMR, 1999–2020 and 2021–2024

| **Category** | **Period** | **Deaths** | **% of overall deaths** |
| --- | --- | --- | --- |
| Overall | 1999-2024 | 296,691 | 100.00% |
| Female | 1999-2024 | 133,212 | 44.90% |
| Male | 1999-2024 | 163,479 | 55.10% |
| **Age Groups** | | | |
| 25-34 years | 1999-2024 | 304 | 0.10% |
| 35-44 years | 1999-2024 | 1,282 | 0.40% |
| 45-54 years | 1999-2024 | 5,140 | 1.70% |
| 55-64 years | 1999-2024 | 17,829 | 6.00% |
| 65-74 years | 1999-2024 | 46,497 | 15.70% |
| 75-84 years | 1999-2024 | 102,910 | 34.70% |
| 85+ years | 1999-2024 | 122,698 | 41.40% |
| **Race/Ethnicity** | | | |
| American Indian or Alaska Native | 1999-2024 | 1,563 | 0.50% |
| Asian or Pacific Islander | 1999-2024 | 9,629 | 3.20% |
| Black or African American | 1999-2024 | 35,284 | 11.90% |
| White | 1999-2024 | 232,118 | 78.20% |
| Hispanic or Latino | 1999-2024 | 17,161 | 5.80% |
| **Census Region** | | | |
| Midwest | 1999-2024 | 63,254 | 21.30% |
| Northeast | 1999-2024 | 45,351 | 15.30% |
| South | 1999-2024 | 119,733 | 40.40% |
| West | 1999-2024 | 68,353 | 23.00% |
| **Urbanization** | | | |
| Metropolitan | 1999-2020 | 202,497 | 68.30% |
| Nonmetropolitan | 1999-2020 | 52,253 | 17.60% |
| **Place of Death** | | | |
| Medical Facility - Inpatient | 1999-2024 | 152,417 | 51.30% |
| Medical Facility - Outpatient or ER | 1999-2024 | 5,943 | 2.00% |
| Medical Facility - Dead on Arrival | 1999-2024 | 390 | 0.10% |
| Medical Facility - Status unknown | 1999-2024 | 333 | 0.10% |
| Decedent's home | 1999-2024 | 26,719 | 9.00% |
| Hospice facility | 1999-2024 | 14,750 | 5.00% |
| Nursing home/long term care | 1999-2024 | 87,803 | 29.60% |
| Other | 1999-2024 | 8,014 | 2.70% |
| Place of death unknown | 1999-2024 | 557 | 0.20% |

**Table S1.** Total deaths and percentage distribution by study variables

| **Year** | **Overall** | **Male** | **Female** |
| --- | --- | --- | --- |
| 1999 | 9.2 | 13.38 | 6.79 |
| 2000 | 9.11 | 13.27 | 6.68 |
| 2001 | 8.92 | 12.83 | 6.76 |
| 2002 | 8.58 | 12.45 | 6.48 |
| 2003 | 7.88 | 11.33 | 6.02 |
| 2004 | 6.99 | 10.2 | 5.28 |
| 2005 | 6.39 | 8.96 | 4.86 |
| 2006 | 5.82 | 8.12 | 4.33 |
| 2007 | 5.49 | 7.59 | 4.07 |
| 2008 | 4.73 | 6.64 | 3.56 |
| 2009 | 4.42 | 6.11 | 3.36 |
| 2010 | 4.57 | 6.42 | 3.39 |
| 2011 | 4.46 | 6.25 | 3.31 |
| 2012 | 4.13 | 5.81 | 2.99 |
| 2013 | 4.02 | 5.61 | 2.94 |
| 2014 | 4.04 | 5.58 | 2.95 |
| 2015 | 4.15 | 5.76 | 3.03 |
| 2016 | 4.05 | 5.6 | 2.95 |
| 2017 | 3.98 | 5.55 | 2.9 |
| 2018 | 3.78 | 5.22 | 2.72 |
| 2019 | 3.67 | 5.03 | 2.64 |
| 2020 | 3.91 | 5.43 | 2.75 |
| 2021 | 4.17 | 5.73 | 2.87 |
| 2022 | 3.95 | 5.52 | 2.65 |
| 2023 | 3.72 | 5.1 | 2.55 |
| 2024 | 3.55 | 4.92 | 2.46 |

**Table S2.** Annual AAMR by overall mortality and sex, 1999-2024

| **Year** | **White** | **Black or African American** | **American Indian or Alaska Native** | **Asian or Pacific Islander** | **Hispanic or Latino** |
| --- | --- | --- | --- | --- | --- |
| 1999 | 8.89 | 12.51 | 11.94 | 9.11 | 8.24 |
| 2000 | 8.79 | 12.94 | 9.89 | 8.87 | 8.19 |
| 2001 | 8.56 | 12.86 | 10.72 | 8.55 | 8.07 |
| 2002 | 8.39 | 12.05 | 10.21 | 8.36 | 7.37 |
| 2003 | 7.77 | 11.21 | 7.72 | 7.61 | 7.17 |
| 2004 | 6.83 | 9.65 | 9.05 | 6.77 | 5.99 |
| 2005 | 6.21 | 8.86 | 6.19 | 5.99 | 5.14 |
| 2006 | 5.67 | 7.75 | 7.47 | 5.33 | 5.13 |
| 2007 | 5.32 | 7.32 | 7.38 | 4.81 | 4.4 |
| 2008 | 4.62 | 6.09 | 5.4 | 4.59 | 3.94 |
| 2009 | 4.82 | 6.04 | 6.85 | 4.59 | 3.87 |
| 2010 | 4.54 | 6.14 | 6.03 | 4.46 | 4.18 |
| 2011 | 4.42 | 5.81 | 7.37 | 4.41 | 4.05 |
| 2012 | 4.14 | 5.43 | 4.39 | 4.17 | 3.93 |
| 2013 | 3.87 | 5.3 | 3.87 | 3.98 | 3.67 |
| 2014 | 3.93 | 5.36 | 5.01 | 3.93 | 3.73 |
| 2015 | 4 | 5.59 | 3.91 | 3.89 | 3.71 |
| 2016 | 3.9 | 5.4 | 6.03 | 3.82 | 3.74 |
| 2017 | 3.84 | 5.33 | 4.2 | 3.78 | 3.48 |
| 2018 | 3.69 | 4.86 | 4.54 | 3.48 | 3.4 |
| 2019 | 3.57 | 5.28 | 3.92 | 3.64 | 3.35 |
| 2020 | 3.69 | 6.04 | 4.11 | 3.37 | 3.63 |
| 2021 | 3.91 | 6.33 | 5.04 | 3.67 | 3.71 |
| 2022 | 3.77 | 5.87 | 2.87 | 3.53 | 3.82 |
| 2023 | 3.58 | 5.51 | 2.71 | 3.44 | 3.62 |
| 2024 | 3.23 | 5.64 | 3.95 | 3.34 | 3.51 |

**Table S3.** Annual AAMR by race and ethnicity, 1999–2024

| **Year** | **Northeast** | **Midwest** | **South** | **West** |
| --- | --- | --- | --- | --- |
| 1999 | 6.82 | 8.74 | 10.62 | 9.89 |
| 2000 | 6.82 | 8.57 | 10.67 | 9.5 |
| 2001 | 6.62 | 8.42 | 10.36 | 9.63 |
| 2002 | 6.5 | 8.44 | 9.73 | 9.38 |
| 2003 | 5.88 | 7.8 | 8.91 | 9.05 |
| 2004 | 5.46 | 6.99 | 7.75 | 8.04 |
| 2005 | 4.86 | 6.77 | 6.98 | 6.73 |
| 2006 | 4.49 | 6.08 | 6.21 | 6.36 |
| 2007 | 3.87 | 5.69 | 5.84 | 6.19 |
| 2008 | 3.6 | 4.95 | 4.94 | 5.42 |
| 2009 | 3.13 | 4.62 | 4.66 | 5.16 |
| 2010 | 3.28 | 4.78 | 4.9 | 5.27 |
| 2011 | 3.23 | 4.63 | 4.63 | 5.17 |
| 2012 | 3.16 | 4.15 | 4.32 | 4.66 |
| 2013 | 3.1 | 4.02 | 4.21 | 4.57 |
| 2014 | 3.01 | 3.96 | 4.32 | 4.67 |
| 2015 | 3.41 | 3.97 | 4.35 | 4.72 |
| 2016 | 2.94 | 4.04 | 4.17 | 4.76 |
| 2017 | 2.89 | 3.98 | 4.3 | 4.53 |
| 2018 | 2.76 | 3.58 | 4.18 | 4.12 |
| 2019 | 2.39 | 3.46 | 4.23 | 3.98 |
| 2020 | 2.64 | 3.79 | 4.55 | 4.12 |
| 2021 | 2.86 | 3.95 | 4.78 | 4.26 |
| 2022 | 2.79 | 3.65 | 4.43 | 4.48 |
| 2023 | 2.5 | 3.52 | 4.2 | 4.1 |
| 2024 | 2.41 | 3.26 | 3.88 | 4.13 |

**Table S4.** Annual AAMR by U.S. Census region, 1999–2024

| **Year** | **Metropolitan** | **Nonmetropolitan** |
| --- | --- | --- |
| 1999 | 9.02 | 9.96 |
| 2000 | 8.98 | 9.62 |
| 2001 | 8.77 | 9.63 |
| 2002 | 8.47 | 9.55 |
| 2003 | 7.82 | 8.91 |
| 2004 | 6.92 | 8.03 |
| 2005 | 6.21 | 7.45 |
| 2006 | 5.69 | 6.52 |
| 2007 | 5.29 | 6.46 |
| 2008 | 4.54 | 5.7 |
| 2009 | 4.25 | 5.21 |
| 2010 | 4.44 | 5.53 |
| 2011 | 4.29 | 5.37 |
| 2012 | 3.97 | 4.96 |
| 2013 | 3.89 | 4.75 |
| 2014 | 3.9 | 4.65 |
| 2015 | 4.03 | 4.91 |
| 2016 | 3.91 | 4.64 |
| 2017 | 3.88 | 4.58 |
| 2018 | 3.71 | 4.39 |
| 2019 | 3.58 | 4.04 |
| 2020 | 3.8 | 4.42 |

**Table S5.** Annual AAMR by urbanization status, 1999–2020

| **Year** | **25-34 years** | **35-44 years** | **45-54 years** | **55-64 years** | **65-74 years** | **75-84 years** | **85+ years** |
| --- | --- | --- | --- | --- | --- | --- | --- |
| 1999 | Unreliable | 0.1 | 0.39 | 2.32 | 12.55 | 51.45 | 163.53 |
| 2000 | Unreliable | 0.1 | 0.48 | 2.48 | 12.18 | 52.38 | 157.04 |
| 2001 | Unreliable | 0.14 | 0.42 | 2.49 | 12.11 | 51.27 | 157.78 |
| 2002 | 0.03 | 0.12 | 0.51 | 2.34 | 11.54 | 49.12 | 154.85 |
| 2003 | 0.03 | 0.08 | 0.48 | 2.09 | 11.09 | 46.01 | 138.85 |
| 2004 | Unreliable | 0.11 | 0.54 | 2.04 | 10.86 | 39.87 | 125.59 |
| 2005 | Unreliable | 0.1 | 0.46 | 2.01 | 10.18 | 37.19 | 112.71 |
| 2006 | Unreliable | 0.1 | 0.51 | 1.85 | 9.72 | 34.65 | 103.37 |
| 2007 | Unreliable | 0.11 | 0.43 | 1.75 | 9.38 | 32.1 | 95.08 |
| 2008 | Unreliable | 0.1 | 0.45 | 1.54 | 8.24 | 30.54 | 84.17 |
| 2009 | Unreliable | 0.08 | 0.47 | 1.47 | 7.78 | 29.27 | 75.88 |
| 2010 | Unreliable | 0.09 | 0.46 | 1.48 | 7.78 | 29.76 | 81.24 |
| 2011 | Unreliable | 0.11 | 0.43 | 1.41 | 7.42 | 29.7 | 75.3 |
| 2012 | Unreliable | 0.1 | 0.44 | 1.39 | 6.81 | 28.51 | 67.09 |
| 2013 | Unreliable | 0.08 | 0.4 | 1.34 | 6.41 | 27.83 | 66.5 |
| 2014 | 0.03 | 0.1 | 0.42 | 1.35 | 6.5 | 27.44 | 66.71 |
| 2015 | 0.02 | 0.11 | 0.49 | 1.45 | 6.37 | 27.89 | 69.78 |
| 2016 | 0.03 | 0.1 | 0.51 | 1.42 | 6 | 28.22 | 68.1 |
| 2017 | 0.03 | 0.1 | 0.56 | 1.53 | 5.72 | 27.91 | 67.24 |
| 2018 | 0.03 | 0.15 | 0.56 | 1.57 | 5.53 | 26.56 | 60.53 |
| 2019 | 0.03 | 0.15 | 0.6 | 1.7 | 5.16 | 25.99 | 58.55 |
| 2020 | 0.05 | 0.18 | 0.7 | 1.93 | 5.33 | 27.58 | 61.3 |
| 2021 | 0.04 | 0.24 | 0.78 | 2.19 | 5.55 | 29.25 | 62.03 |
| 2022 | 0.03 | 0.22 | 0.82 | 2.24 | 5.58 | 27.96 | 57.31 |
| 2023 | 0.04 | 0.22 | 0.74 | 2.04 | 5.01 | 26.4 | 54.97 |
| 2024 | 0.04 | 0.22 | 0.83 | 2.14 | 4.84 | 24.91 | 52.51 |

**Table S6.** Annual crude mortality rates by age group, 1999–2024

| **Place of Death** | **Deaths** | **% of total deaths** |
| --- | --- | --- |
| Medical Facility - Inpatient | 152,417 | 51.30% |
| Medical Facility - Outpatient or ER | 5,943 | 2.00% |
| Medical Facility - Dead on Arrival | 390 | 0.10% |
| Medical Facility - Status unknown | 333 | 0.10% |
| Decedent's home | 26,719 | 9.00% |
| Hospice facility | 14,750 | 5.00% |
| Nursing home/long term care | 87,803 | 29.60% |
| Other | 8,014 | 2.70% |
| Place of death unknown | 557 | 0.20% |

**Table S7.** Place-of-death distribution, 1999–2024

| **State** | **AAMR 1999-2020** | **AAMR 2021-2024** |
| --- | --- | --- |
| Alabama | 5.45 | 3.6 |
| Alaska | 7.27 | 5.34 |
| Arizona | 3.85 | 2.12 |
| Arkansas | 4.08 | 2.95 |
| California | 5.78 | 4.3 |
| Colorado | 5.13 | 4.12 |
| Connecticut | 4.6 | 3.66 |
| Delaware | 5.49 | 3.37 |
| District of Columbia | 7.78 | 6.63 |
| Florida | 3.92 | 4.47 |
| Georgia | 4.94 | 3.13 |
| Hawaii | 7.03 | 3.16 |
| Idaho | 6.74 | 4.6 |
| Illinois | 4.82 | 2.83 |
| Indiana | 5.68 | 5.01 |
| Iowa | 6.63 | 3.25 |
| Kansas | 4.93 | 4.1 |
| Kentucky | 6.58 | 4.59 |
| Louisiana | 3.95 | 2.45 |
| Maine | 4.98 | 0.49 |
| Maryland | 5.85 | 5.09 |
| Massachusetts | 6.18 | 4.39 |
| Michigan | 4.25 | 2.94 |
| Minnesota | 4.66 | 2.84 |
| Mississippi | 4.97 | 3.87 |
| Missouri | 6.23 | 3.9 |
| Montana | 6.07 | 3.62 |
| Nebraska | 5.91 | 3.86 |
| Nevada | 3.95 | 4.08 |
| New Hampshire | 4.6 | 3.66 |
| New Jersey | 4.31 | 3.07 |
| New Mexico | 3.7 | 1.96 |
| New York | 2.27 | 1.58 |
| North Carolina | 7.23 | 3.39 |
| North Dakota | 7.32 | 4.05 |
| Ohio | 6.15 | 4.02 |
| Oklahoma | 6.98 | 4.39 |
| Oregon | 6.68 | 5.1 |
| Pennsylvania | 5.08 | 3.52 |
| Rhode Island | 6.18 | 4.39 |
| South Carolina | 7.93 | 6.27 |
| South Dakota | 6.81 | 4.45 |
| Tennessee | 8.72 | 4.96 |
| Texas | 7.02 | 5.09 |
| Utah | 5.42 | 3.4 |
| Vermont | 8.17 | 3.66 |
| Virginia | 4.94 | 3.37 |
| Washington | 9.31 | 7.22 |
| West Virginia | 5.49 | 1.87 |
| Wisconsin | 5.04 | 3.93 |
| Wyoming | 4.35 | 3.96 |

**Table S8.** State-level AAMR, 1999–2020 and 2021–2024
